# Passion Fruit Chlorotic Mottle Virus: Molecular Characterization of a New Divergent Geminivirus in Brazil

**DOI:** 10.3390/v10040169

**Published:** 2018-04-02

**Authors:** Rafaela S. Fontenele, Rayane A. Abreu, Natalia S. Lamas, Dione M. T. Alves-Freitas, Andreza H. Vidal, Raul R. Poppiel, Fernando L. Melo, Cristiano Lacorte, Darren P. Martin, Magnolia A. Campos, Arvind Varsani, Simone G. Ribeiro

**Affiliations:** 1 Embrapa Recursos Genéticos e Biotecnologia, Brasília, DF 70770-017, Brazil; rfontene@asu.edu (R.S.F.); rayannyalexandre@gmail.com (R.A.A.); nslamas@gmail.com (N.S.L.); dionebio@gmail.com (D.M.T.A.-F.); andrezactg@hotmail.com (A.H.V.); raulpoppiel@gmail.com (R.R.P.); cristiano.lacorte@embrapa.br (C.L.); 2 The Biodesign Center for Fundamental and Applied Microbiomics, Center for Evolution and Medicine, School of Life Sciences, Arizona State University, Tempe, AZ 85287, USA; 3 Centro de Educação e Saúde, Universidade Federal de Campina Grande, Cuité, PB 58175-000, Brazil; magnoliacp@gmail.com; 4 Departmento de Biologia Celular, Campus Universitário Darcy Ribeiro, Universidade de Brasília, Brasília, DF 70910-900, Brazil; flucasmelo@gmail.com; 5 Computational Biology Group, Institute of Infectious Disease and Molecular Medicine, Faculty of Health Sciences, University of Cape Town, Observatory 7925, South Africa; darrenpatrickmartin@gmail.com; 6 Structural Biology Research Unit, Department of Integrative Biomedical Sciences, University of Cape Town, Observatory, Cape Town 7701, South Africa

**Keywords:** geminivirus, passion fruit, *Passiflora* sp.

## Abstract

Brazil is one of the major passion fruit producers worldwide. Viral diseases are among the most important constraints for passion fruit production. Here we identify and characterize a new passion fruit infecting-virus belonging to the family *Geminiviridae*: passion fruit chlorotic mottle virus (PCMoV). PCMoV is a divergent geminivirus unlike previously characterized passion fruit-infecting geminiviruses that belonged to the genus *Begomovirus*. Among the presently known geminiviruses, it is most closely related to, and shares ~62% genome-wide identity with citrus chlorotic dwarf associated virus (CCDaV) and camelia chlorotic dwarf associated virus (CaCDaV). The 3743 nt PCMoV genome encodes a capsid protein (CP) and replication-associated protein (Rep) that respectively share 56 and 60% amino acid identity with those encoded by CaCDaV. The CPs of PCMoV, CCDaV, and CaCDaV cluster with those of begomovirus whereas their Reps with those of becurtoviruses. Hence, these viruses likely represent a lineage of recombinant begomo-like and becurto-like ancestral viruses. Furthermore, PCMoV, CCDaV, and CaCDaV genomes are ~12–30% larger than monopartite geminiviruses and this is primarily due to the encoded movement protein (MP; 891–921 nt) and this MP is most closely related to that encoded by the DNA-B component of bipartite begomoviruses. Hence, PCMoV, CCDaV, and CaCDaV lineage of viruses may represent molecules in an intermediary step in the evolution of bipartite begomoviruses (~5.3 kb) from monopartite geminiviruses (~2.7–3 kb). An infectious clone of PCMoV systemically infected *Nicotiana benthamina*, *Arabidopsis thaliana*, and *Passiflora edulis*.

## 1. Introduction

Passion fruit is an economically important food crop in many tropical and sub-tropical regions of the world. In Brazil, where passion fruit likely originated, viral diseases are a major constraint for passion fruit production. Viruses infecting passion fruit in other parts of the world generally have single-stranded RNA genomes and belong to the families *Betaflexiviridae* (genus: *Carlavirus*), *Bromoviridae* (genus: *Cucumovirus*), *Potyviridae* (genus: *Potyvirus*), and *Virgaviridae* (genus: *Tobamovirus*). Passion fruit woodiness disease (PWD) is the most economically relevant disease of passion fruit and is caused by viruses classified in one of three potyvirus species, *Passion fruit woodiness virus* [1], *East Asian passiflora virus* [2], and *Cowpea aphid-borne mosaic virus* [3]. In Brazil, PWD is primarily attributed to cowpea aphid-borne mosaic virus (CABMV) which is widespread and is, to date, the only potyvirus to be identified associated with passion fruit in the country [3,4]. The only known passion fruit infecting viruses that have single-stranded DNA (ssDNA) genomes belong to the genus *Begomovirus* (family *Geminiviridae*).

Geminiviruses infect many other important cultivated and non-cultivated plants worldwide. Their circular ssDNA genomes are encapsidated in twined icosahedral (geminate) particles. There are nine recognized geminivirus genera: *Becurtovirus*, *Begomovirus*, *Capulavirus*, *Curtovirus*, *Eragrovirus*, *Glabovirus*, *Mastrevirus*, *Topocuvirus*, and *Turncurtovirus* [5,6]. Besides these genera, there are also several other highly divergent geminivirus species that have not yet be assigned to a genus. These unassigned viruses include citrus chlorotic dwarf associated virus (CCDaV) [7], mulberry mosaic dwarf associated virus (MMDaV) [8], apple geminivirus (AGmV) [9], grapevine geminivirus A (GGmV A) [10], tomato associated geminivirus 1 (TaGmV 1) [11], tomato apical leaf curl virus (TALCV) [12], camelia chlorotic dwarf associated virus (CaCDaV) [13], Limeum africanum associated virus (LaaV), Polygala garcini associated virus (PgaV), and Juncus maritimus associated virus (JmaV) [14].

The discovery of most of these novel divergent geminiviruses has been facilitated by recent methodological advances in both nucleotide sequencing and viral genome enrichment techniques [15]. Prior to these advances, the overwhelming focus of past virus discovery efforts was the detection and sequencing of viruses that cause recognizable diseases in cultivated plants. However, high throughput sequencing (HTS) and viral genome enrichment techniques such as rolling circle amplification (RCA) [16] have enabled plant viral metagenomics approaches capable of simultaneously detecting and partially characterizing thousands of viruses within hundreds of different plant samples, reviewed in Roossinck, Martin, and Roumagnac [15]. Such approaches are now revealing the true extent of plant virus diversity within both managed and natural environments [17].

Here we describe the characterization of a divergent new geminivirus found co-infecting a diseased passion fruit plant together with CABMV in the Midwest region of Brazil—state of Mato Grosso do Sul. We show that this new highly divergent geminivirus is most closely related to the unassigned geminiviruses CCDaV and CaCDaV and was able to infect *Nicotiana benthamina*, *Arabidopsis thaliana*, and *Passiflora edulis*. Based on the host and the symptoms presented, the new geminivirus has been tentatively named passion fruit chlorotic mottle virus (PCMoV; Supplementary Figure S1).

## 2. Materials and Methods

### 2.1. Sample Collection and Processing

As part of routine plant-infecting virus surveillance in the Midwest region of Brazil, samples from a variety of plant species were collected between 2013 and 2015. The samples included a *Passiflora* sp. sample collected in the state of Mato Grosso do Sul in the year 2014. This sample exhibited symptoms consistent with viral infection including chlorosis, crinkling, and leaf deformation. Total DNA was extracted from the sample using the CTAB method [18] and circular DNA was amplified by rolling circle amplification (RCA) using Φ29 DNA polymerase (New England Biolabs Inc., Ipswich, MA, USA). Double-strand RNA was also extracted from the *Passiflora* sp. sample using micro-spin column method as described by [19,20] but modified slightly by using medium cellulose fiber C6288 (Sigma-Aldrich Inc., St. Louis, MO, USA).

### 2.2. High Throughput Sequencing and Genome Assembly

The RCA products and the RNA molecules were sequenced separately on an Illumina HiSeq 2500 platform (2 × 125 paired-end) at Macrogen Inc., Seoul, Korea. The reads obtained by the high throughput sequencing were de novo assembled using ABySS 1.9 [21] with a k-mer of 64. The assembled contigs were analyzed by BLASTx [22] against a viral sequence database.

Based on the de novo assembled contigs of the sequenced RCA DNA products, a set of abutting primers spanning a *Bam*HI site was designed (PF_BamHIF 5′-GGA TCC CGC TCA AGT GAT G-3′ and PF_BamHIR 5′-ATC GGC GTA ACA GCA TAA A-3′) to recover a potentially full-length geminivirus-like genome sequence from the analyzed passion fruit sample. The primers were used with KAPA HiFi Hotstart DNA polymerase (Roche Molecular Systems, Inc, Pleasanton, CA, USA) with the thermal cycling conditions: 98 °C for 3 min, 25 cycles of 98 °C for 15 s, 60 °C for 15 s, 72 °C for 3 min, and a final extension of 72 °C for 4 min to recover the full genome.

Based on the RNA de novo assembled contigs, two primer pairs were designed CABMV_M1MX_473_F 5′-GAC TTC AAC CAA CTT GAC ACT AGT G-3′/CABMV_M1MX_1011_R 5′-CAA GCT GCA CAA CTT GTT CTA AAA C-3′ and CABMV_M1MX_3726_F 5′-GAG ACA CAA GCC AAA ACA CAA AAT C-3′/CABMV_M1MX_5039_R 5′-CGT TGC TAC AAA TTC TGG TAT CTC C-3′ to amplify a 540 nt and 1311 nt region, respectively, of the potyvirus CABMV. The cDNA synthesis and polymerase chain reaction (PCR) amplifications were performed using the SuperScript™ III One-Step RT-PCR System with Platinum™ *Taq* DNA Polymerase (ThermoFisher Scientific, Waltham, MA, USA).

The PCR products were resolved in an agarose gel and the amplicon of the expected size was excised, gel purified and cloned in the plasmid pJET 1.2 (ThermoFisher Scientific). Cloned DNA fragments were Sanger sequenced by primer walking at Macrogen Inc. (Seoul, Korea). Sequence reads were assembled and analyzed in Geneious v11 [23].

### 2.3. Phylogenetic and Pairwise Identity Analyses

A geminivirus-like genome determined in this study together with 30 representative sequences of the nine established geminivirus genera and ten other geminivirus genome sequences that have not yet been assigned to any genera were aligned using MAFFT v7 [24]. The movement protein encoding regions of the genomes were removed to yield a better alignment. This alignment was then used to infer a neighbor-joining phylogenetic tree (with a Jukes-Cantor nucleotide substitution model and 1000 bootstrap replicates) using MEGA5 [25]. Branches with bootstrap support <60% were collapsed using TreeGraph2 [26] and the tree was midpoint rooted.

The same representative sequences used in the genome nucleotide sequence analysis were used to create an alignment with the inferred capsid protein (CP) and replication associated protein (Rep) amino acid sequences using MUSCLE v3.8.31 [27]. The CP and Rep amino acid sequence alignments were used to infer Maximum-likelihood phylogenetic trees with the rtREV+G+I+F and rtREV+G+I amino acid substitution models (inferred as best fit models using ProtTest [28], respectively, and the approximate likelihood ratio test (aLRT) used to determine degrees of branch support. Branches from the CP and Rep phylogenetic trees with aLRT support <0.8 were collapsed using TreeGraph2 [26]. The trees were rooted with the CP and Rep amino acid sequences of the genomoviruses Sclerotinia sclerotiorum hypovirulence associated DNA virus 1 (KM598384) and MSSI2.225 virus (LK931485) as outgroup sequences, especially due to the fact that the Reps of genomoviruses are most closely related to those of geminiviruses.

Begomovirus DNA-B sequences were downloaded from GenBank and the *mp* ORFs were extracted and translated. Movement proteins (MPs) of DNA-B were grouped using SDT v1.2 [29] based on 75% amino acid identity and a representative of each group or singleton was then aligned with the MPs of the new geminivirus from this study and, those of CCDaV and CaCDaV using MUSCLE [27]. A Maximum likelihood tree with the rtREV+G amino acid substitution model, inferred as best fit models using ProtTest [28], was inferred using PHYML 3.0 [30] and the tree was midpoint rooted. Braches with <0.8 aLRT support were collapsed using TreeGraph2 [26].

All pairwise identities (genome-wide, CP, MP and Rep) were determined using SDT v1.2 [29].

### 2.4. Infectivity Assays

The cloned geminivirus-like genome was excised from pJET 1.2 by restriction with *Bam*HI (ThermoFisher Scientific, ‎Waltham, MA, USA) and five micrograms of the linearized virus genome was re-ligated, precipitated on tungsten particles and bombarded into *N. benthamiana* (*n* = 40), *A. thaliana* (*n* = 7), *P. edulis* (*n* = 40) and *P. alata* (*n* = 3) plants using a high pressure device as described by Blawid, et al. [31]. Furthermore, leaves of *N. benthamiana* inoculated by particle bombardment with PCMoV and the original *Passiflora* sp. plant dually infected by the new geminivirus and CABMV were ground up in inoculation buffer (phosphate buffer 10 mM, pH 8.0, 0.01% Na_2_SO_3_, 25 mM EDTA) and the extracts were mechanically inoculated onto carborundum-dusted *N. benthamiana* and *P. edulis* plantlets.

Active infection in the inoculated plants was confirmed by PCR using the abutting primers PF_BamHI F/R and the DNA extracted from non-inoculated new top leaves using CTAB method [18] 15 and 30 days post inoculation as a template. Primers CABMV_M1MX_473_F and CABMV_M1MX_1011_R were used to confirm CABMV transmission. The amplicons were resolved in a 0.7% agarose gel, excised, gel purified, cloned in pJET 1.2 (ThermoFisher Scientific, ‎Waltham, MA, USA), and Sanger sequenced at Macrogen Inc. (Seould, South Korea).

Infection by the new geminivirus-like virus was further confirmed using Southern blot hybridization. Total DNA from infected plants (15 µg) in addition to the total DNA of the original *Passiflora* sp. plant from which the virus was isolated, used as positive control, were resolved in a 1% agarose gel and then transferred to a nylon membrane Hybond-N+ (GE Healthcare, Pittsburgh, PA, USA). The membrane was hybridized with a αP^32^ dCTP labeled probe specific for the geminivirus-like full genome using the Rediprime II DNA Labeling System (GE Healthcare, Pittsburgh, PA, USA) kit.

## 3. Results and Discussion

### 3.1. Identification of a Novel Geminivirus

A *Passiflora* sp. sample was collected in the state of Mato Grosso do Sul, in the Midwest Brazil, displaying symptoms consistent to viral infection, as part of a plant-infecting virus surveillance. The DNA from the sample was extracted and circular viral genomes amplified using RCA prior to HTS. BLASTx searches of the de novo assembled contigs from the RCA HTS against the The National Center for Biotechnology Information (NCBI) GenBank RefSeq database revealed a 1969 nt contig with a high degree of similarity to CCDaV (31% query coverage, 62% identity with e-value of 3 × 10^−89^).

**Figure 1:**
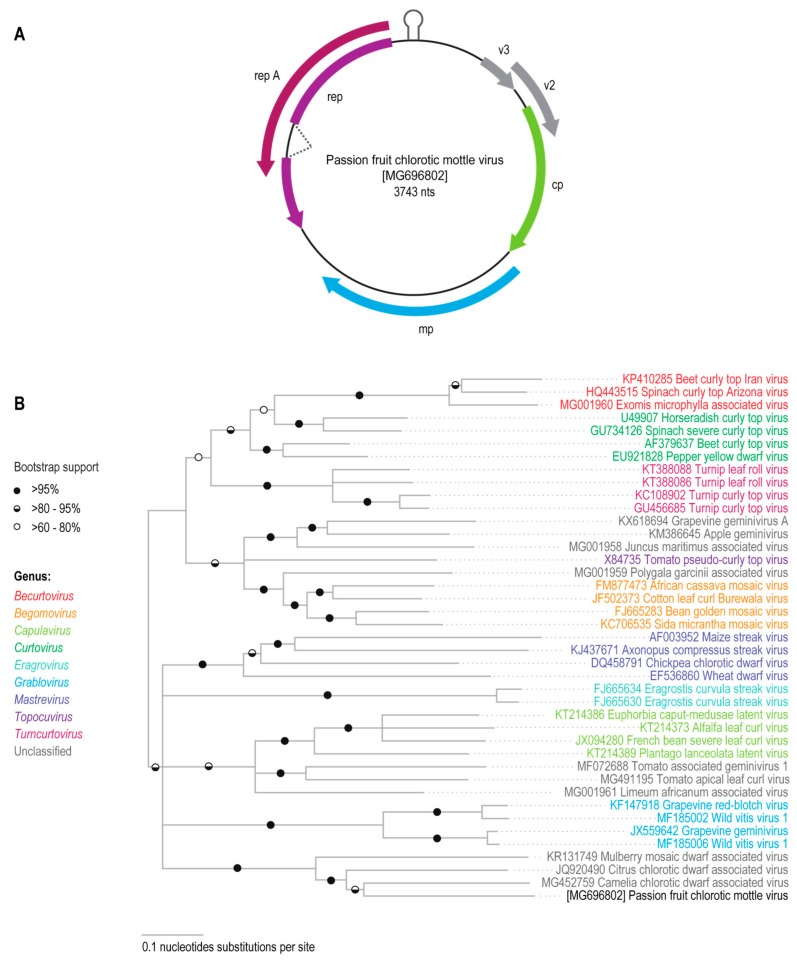
(**A**) Genome organization of passion fruit chlorotic mottle virus (PCMoV); (**B**) Midpoint rooted Neighbor-joining tree with Jukes-Cantor substitution model and 1000 bootstraps iterations for the genomes (or DNA-A) sequences of representative geminiviruses and PCMoV identified in this study. Branches with <60% bootstrap support were collapsed.

To facilitate the cloning of a potentially full-length geminivirus genome, this 1969 nucleotide long geminivirus-like sequence was used to design abutting primers that would enable the recovery a circular viral genomic DNA molecule from the *Passiflora* sp. sample. Amplification with these primers yielded a ~3700 nt amplification product that was subsequently cloned and sequenced. This geminivirus-like genomic sequence (3743 nt) contains a putative stem-loop structure formed by CG-rich inverted repeats flanking the nonanucleotide motif, “TAATATTAC”, which is highly conserved at the origin of virion strand replication in geminivirus genomes. The genome also contains six open reading frames (ORFs) that could potentially encode proteins greater than 76 amino acids in length. The complementary strand of the genome potentially encodes geminivirus-like RepA and/or Rep proteins. By analogy with other geminiviruses, these Rep and RepA proteins could potentially be expressed from an alternatively spliced complementary strand transcript [32]. The virion sense genome strand potentially encodes a CP, MP, and two other small hypothetical proteins, referred to here as V2 and V3 (Figure 1A).

Pairwise identity comparisons of the new geminivirus-like sequence with those of representative geminiviruses indicated that it shares the highest degree of identity (62%) with two of the divergent geminiviruses that have remained unassigned to a genus: CCDaV and CaCDaV (Supplementary Data 1). Pairwise identity analysis of the CP and Rep amino acid sequences that are potentially encoded by the geminivirus-like genome revealed that these proteins respectively share 56% and 60% identity with those of CaCDaV (Supplementary Data 1).

**Figure 2:**
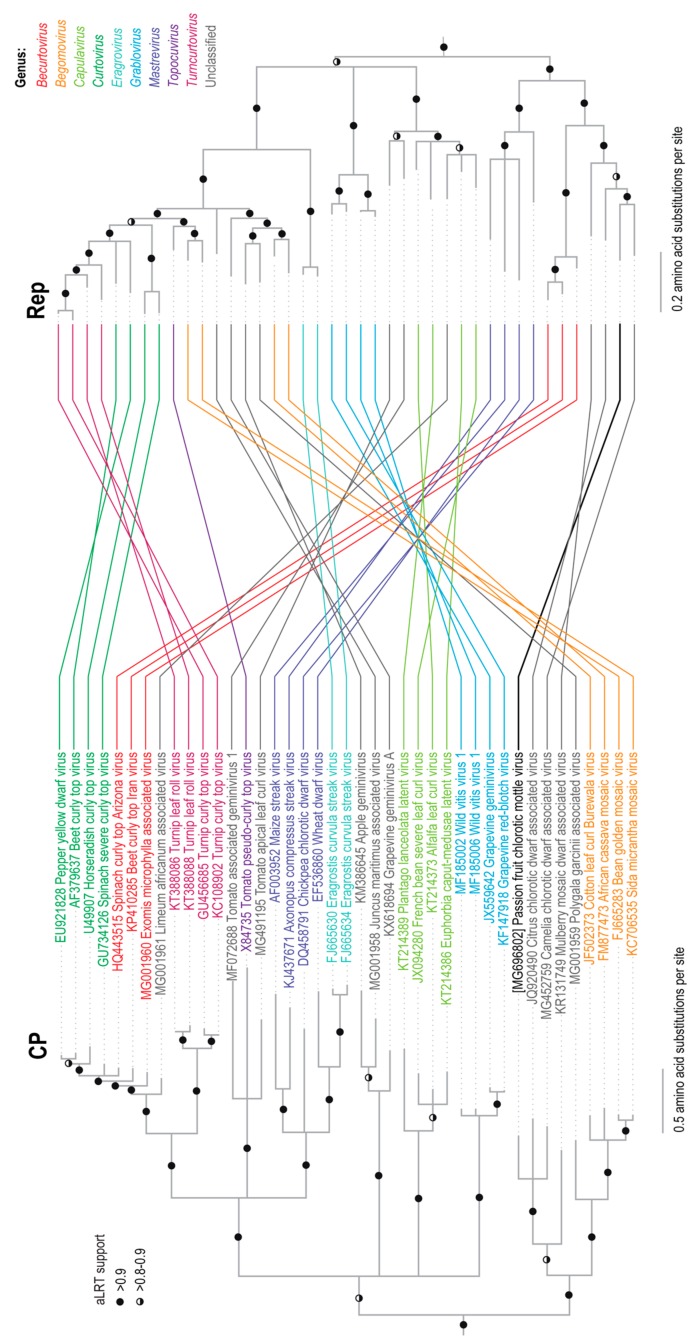
Maximum likelihood phylogenetic tree inferred using the substitution models rtREV+G+I+F and rtREV+G+I for capsid protein (CP) and Rep amino acid sequences, respectively, with an approximate likelihood ratio test (aLRT) for branch support of representative geminiviruses and PCMoV identified in this study. Both trees are rooted with the CP and Rep amino acid sequences of the genomovirus, Sclerotinia sclerotiorum hypovirulence associated DNA virus 1 (KM598384) and MSSI2.225 virus (LK931485). Branches with <0.8 aLRT branch support have been collapsed.

Phylogenetic analysis of the new geminivirus-like sequence from the *Passiflora* sp. sample together with the full genome (or DNA-A sequences) of other known geminiviruses indicated that the new sequence clusters together with the CCDaV and CaCDaV sequences (Figure 1B). Phylogenetic analysis of the amino acid sequences of the probable Rep and CP proteins encoded by the new geminivirus-like sequence indicated that these also cluster with the corresponding proteins of CCDaV and CaCDaV. It is noteworthy that the phylogenetic placement of the clades containing the new geminivirus sequence, CCDaV, CaCDaV, and MMCaV differed between the CP and Rep trees. The CPs of the new geminivirus sequence, CCDaV, CaCDaV, and MMCaV cluster with the begomovirus CP lineage whereas their Reps cluster with those of becurtoviruses (Figure 2). It thus likely, and perhaps not surprising, that like many geminiviruses this new virus is a recombinant of begomo-like and becurto-like ancestral viruses.

**Figure 3:**
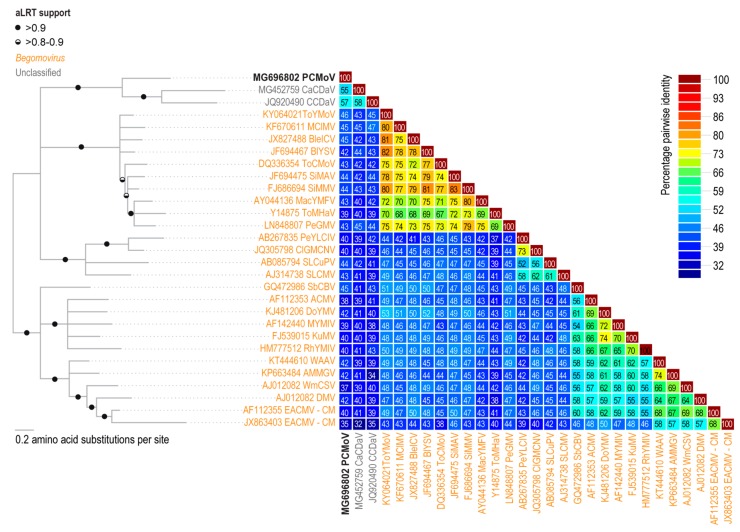
Midpoint rooted Maximum likelihood phylogenetic tree inferred using the substitution model rtREV+G for the MP amino acid sequences and aLRT test for branch support coupled with percentage pairwise identity matrix of the MP amino acid sequences. The sequences used for both analysis are those encoded by the following: African cassava mosaic virus (ACMV), asystasia mosaic Madagascar virus (AMMGV), blainvillea yellow spot virus (BlYSV), blechum interveinal chlorosis virus (BleICV), CaCDaV, CCDaV, clerodendrum golden mosaic China virus (ClGMCNV), deinbollia mosaic virus(DMV), dolichos yellow mosaic virus (DoYMV), East African cassava mosaic virus-Cameroon (EACMV-CM), Kudzu mosaic virus (KuMV), macroptilium yellow mosaic Florida virus (MacYMFV), melon chlorotic mosaic virus (MClMV), mungbean yellow mosaic India virus (MYMIV), PCMoV, pepper golden mosaic virus (PeGMV), pepper yellow leaf curl Indonesia virus (PeLCIV), rhynchosia yellow mosaic India virus (RhYMIV), sida micrantha mosaic virus (SiMMV), sida mosaic Alagoas virus (SiMAV), soybean chlorotic blotch virus (SbCBV), squash leaf curl Philippines virus (SLCuPV), Sri Lankan cassava mosaic virus (SLCMV), tomato chlorotic mottle virus (ToCMoV), tomato mosaic Havana virus (ToMHaV), ToYMoV, watermelon chlorotic stunt virus (WmCSV), and West African asystasia virus (WAAV).

**Figure 4:**
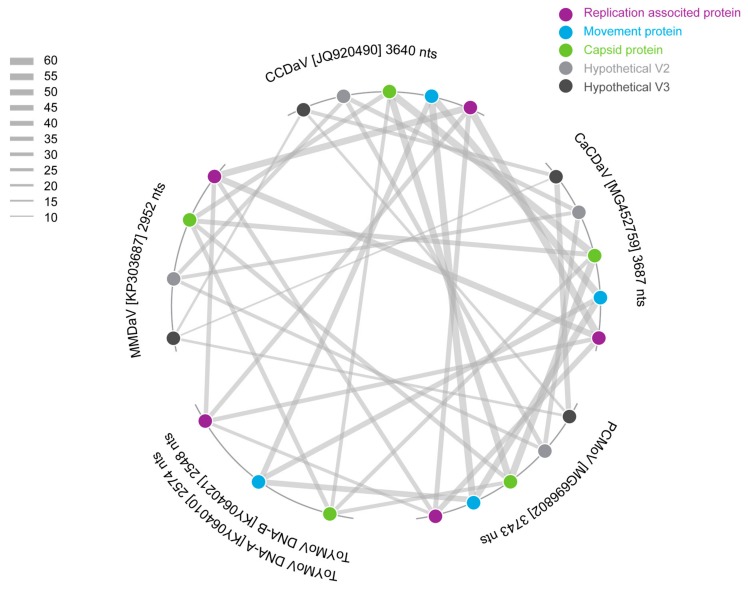
Pairwise identity comparison of the MP, CP, Rep, hypothetical V2 protein and V3 protein of PCMoV, CaCDaV, CCDaV, MMDaV, and ToYMoV.

The pairwise identity analysis of the potentially encoded movement protein (MP) from the new virus indicated that it shares 57% amino acid sequence identity with the potential MP of CCDaV, 55% with that of CaCDaV and 46% with the MP of a bipartite begomovirus, tomato yellow mottle virus (ToYMoV; KY064021) isolated in Costa Rica [33] (Figure 3 and Figure 4). The pairwise identities with other geminiviruses of the predicted amino acid sequences of the Reps and CPs of CCDaV, CaCDaV, and the new geminivirus-like sequence range from 25 to 32% and 30 to 32%, respectively (Figure 4).

To further characterize the genome, a pairwise identity analysis was undertaken with all the ORFs of viruses that are most closely related to the new geminivirus-like sequence and the begomovirus ToYMoV (Figure 4). It is clear from the analysis that the MPs encoded by CCDaV, CaCDaV, and PCMoV share significant amino acid sequence identity (43–46%) with the MP of the bipartite begomovirus ToYMoV (Figure 3 and Figure 4) and other related begomovirus MPs.

### 3.2. CABMV Identified Coinfecting Passion Fruit with the Novel Geminivirus

The dsRNA from the *Passiflora* sp. sample was extracted and submitted to HTS. The de novo assembly of the RNA HTS sequences resulted in a 9874 nt contig encompassing 99.4% of the CABMV genome. This shared 96% identity with a Brazilian CABMV isolate sequence (HQ880243) [34]. To confirm the presence of this virus in the *Passiflora* sp. sample, two primer pairs were designed to amplify CABMV genome fragments of 540 nt and 1311 nt in length. Amplicons of these fragments were cloned and sequenced. The sequences of the clones obtained for the fragments confirmed that CABMV was indeed coinfecting the passion fruit sample together with the new geminivirus-like sequence. Importantly, this is not the first instance where CABMV and a geminivirus have been found coinfecting passion fruit. Also in Brazil, the begomovirus passion fruit leaf distortion virus (PFLDV), was previously found in a passion fruit coinfection with CABMV [35].

### 3.3. Infectivity Assays

The infectivity of the new geminivirus-like sequence in a variety of host species was assessed by particle bombardment and mechanical inoculation approaches. *N. benthamiana* and *P. edulis* were inoculated through both mechanisms while *A. thaliana* and *P. alata* were only inoculated via particle bombardment. Infectivity was confirmed by PCR, Southern blot analysis, and Sanger sequencing. After 15 days only *N. benthamiana* plants displayed symptoms of infection that included chlorotic spots, mottle and a growth impairment that continued to develop until 30 days post inoculation (Supplementary Figure S1). Meanwhile, the other three plant species showed no symptoms at 15 or 30 days after inoculation.

In the particle bombarded plants, infection was assessed by PCR using the abutting primers PF_BamHI F/R in newly emerged leaves from all plants tested. The only tested plant species in which no virus was detected by amplification was *P. alata*.

The rate of infection varied according to the inoculated plants species. Of 40 inoculated *N. benthamiana* plants, 35 were PCR positive for the presence of the geminivirus-like sequence. In *A. thaliana* four out of seven plants were positive for the virus. Infectivity was considerably lower in *P. edulis* plants with only two out of 40 plants being found to be PCR positive for the virus. Other studies have shown similar low infection rates with particle bombardment of begomovirus in *Passiflora* spp. demonstrating that this might be a limitation of the inoculation procedure and not necessarily an indication of low-infectivity of the virus [36]. Nonetheless, it was demonstrated via particle bombardment that the new geminivirus is able to replicate and establish a systemic infection in *N. benthamiana*, *A. thaliana*, and *P. edulis*.

Mild symptoms were observed six months after inoculation in the two successfully infected *P. edulis* plants (Supplementary Figure S1) and virus was still detectable by PCR at that time. Southern blot analysis further confirmed the infection of the inoculated plants (Supplementary Figure S2). The mechanical inoculation was only effective for CABMV as 100% of the inoculated plants became infected. The fact that none of the tested plants was successfully infected with PCMoV by mechanical inoculation indicates that, like other geminiviruses, this new virus is probably not mechanically transmitted. The natural transmission vector for the virus remains unknown. Based on the symptoms observed on the leaves of the two successfully inoculated *P. edulis* plants, we have tentatively named this virus passion fruit chlorotic mottle virus (PCMoV) (Supplementary Figure S1).

PCMoV infectious clone was able to infect *N. benthamiana*, *A. thaliana,* and *P. edulis* via particle bombardment with systemic infection being confirmed by PCR, Southern blotting and sequencing. The passion fruit sample from which this sequence was isolated was co-infected with the common and widespread passion-fruit infecting potyvirus, CABMV. It was therefore not possible to assess whether or not the mild symptoms arising on PCMoV infected *P. edulis* plants resemble those found in PCMoV infected plants in the field.

## 4. Conclusions

Using a high throughput sequencing approach, a novel geminivirus was identified in a Brazilian passion fruit plant. The new geminivirus sequence is 3743 nt in length and potentially encodes six proteins: the RepA, Rep, CP, MP, and two other small proteins. The genome also contains the conserved nonanucleotide motif “TAATATTAC” that is characteristic of geminiviruses. Analysis of the PCMoV genome sequence showed that, among the known geminiviruses, it is most closely related to two of the geminiviruses, CCDaV (3640–3642 nts) and CaCDaV (3687 nts), that are currently unassigned to a genus.

The most intriguing characteristic of the PCMoV, CCDaV, and CaCDaV sequences is that they are between 12% and 30% larger than the genomes of other known monopartite geminiviruses. Their greater size is primarily attributable to the size of their presumed *mp* gene (891–921 nt in size) which is up to three times larger than that of monopartite geminiviruses but is approximately the same size as that of bipartite geminiviruses in the genus *Begomovirus.* However, in bipartite begomoviruses the 714 to 1107 nt long *mp* gene that encodes a protein with detectable homology to the MPs of PCMoV, CCDaV, and CaCDaV is found on the DNA-B molecule. This suggests that PCMoV, CCDaV, and CaCDaV may represent an intermediary step in the evolution of bipartite begomoviruses (with genome sizes of approximately 5.3 kb) from monopartite geminiviruses (with genome sizes that are generally between 2.7 and 3 kb in length).

Geminiviruses in general have geminate particles (~22 × 38 nm) composed of two joined incomplete *T* = 1 icosahedra that packages the 2.6–2.9 kb ssDNA genome [37,38,39]. Subgenomic molecules of geminiviruses are common in infections and Casado et al. [40] showed that ~1.5 kb subgenomic molecules of the maize streak virus (genus *Mastrevirus*) are packaged into single isometric *T* = 1 particles. Furthermore, in a sucrose gradient fraction analyzed by Frischmuth et al. [41] where virions with three joined incomplete *T* = 1 icosahedra were purified, the DNA isolated from this fraction was of higher than normal geminivirus molecular weight DNA. Therefore, results from Casado et al. [40] and Frischmuth et al. [41] suggests that the geminivirus virion assembly is likely driven by genome size. Hence, it is possible that PCMoV, CCDaV, and CaCDaV encode CPs capable of assembling geminate particles with higher plasticity to enable the encapsidation of their larger genomes, or their genomes are packaged into three joined incomplete *T* = 1 icosahedra virions or other possible configurations.

## Supplementary Materials

The following are available online at http://www.mdpi.com/1999-4915/10/4/169/s1, Figure S1: PCMoV infection in host plants: (A) Naturally infected *Passiflora* sp. displays symptoms of chlorosis, crinkling and leaf deformation. Plants inoculated via particle bombardment: (B) *P. edulis* leaf showing chlorotic mottling; (C) *N. benthamiana* plant presenting chlorotic spots and mottle symptoms; and (D) *A. thaliana* is asymptomatic, Figure S2: Southern blot analysis of the total DNA from (A) *N. benthamiana*, (B) *P. edulis*, and (C) *A. thaliana* plants inoculated via particle bombardment with PCMoV, Data S1: Percentage pairwise identity matrix for the nucleotide sequence of the genomes and amino acid sequences of the CP and Rep from representative geminiviruses and passion fruit chlorotic mottle virus using SDT v1.2.
