# Supplementary figures and images for: Passion Fruit Chlorotic Mottle Virus: Molecular Characterization of a New Divergent Geminivirus in Brazil
Source: Viruses. 2018 Apr 2;10(4):169. doi: 10.3390/v10040169 (PMC5923463; doi:10.3390/v10040169)

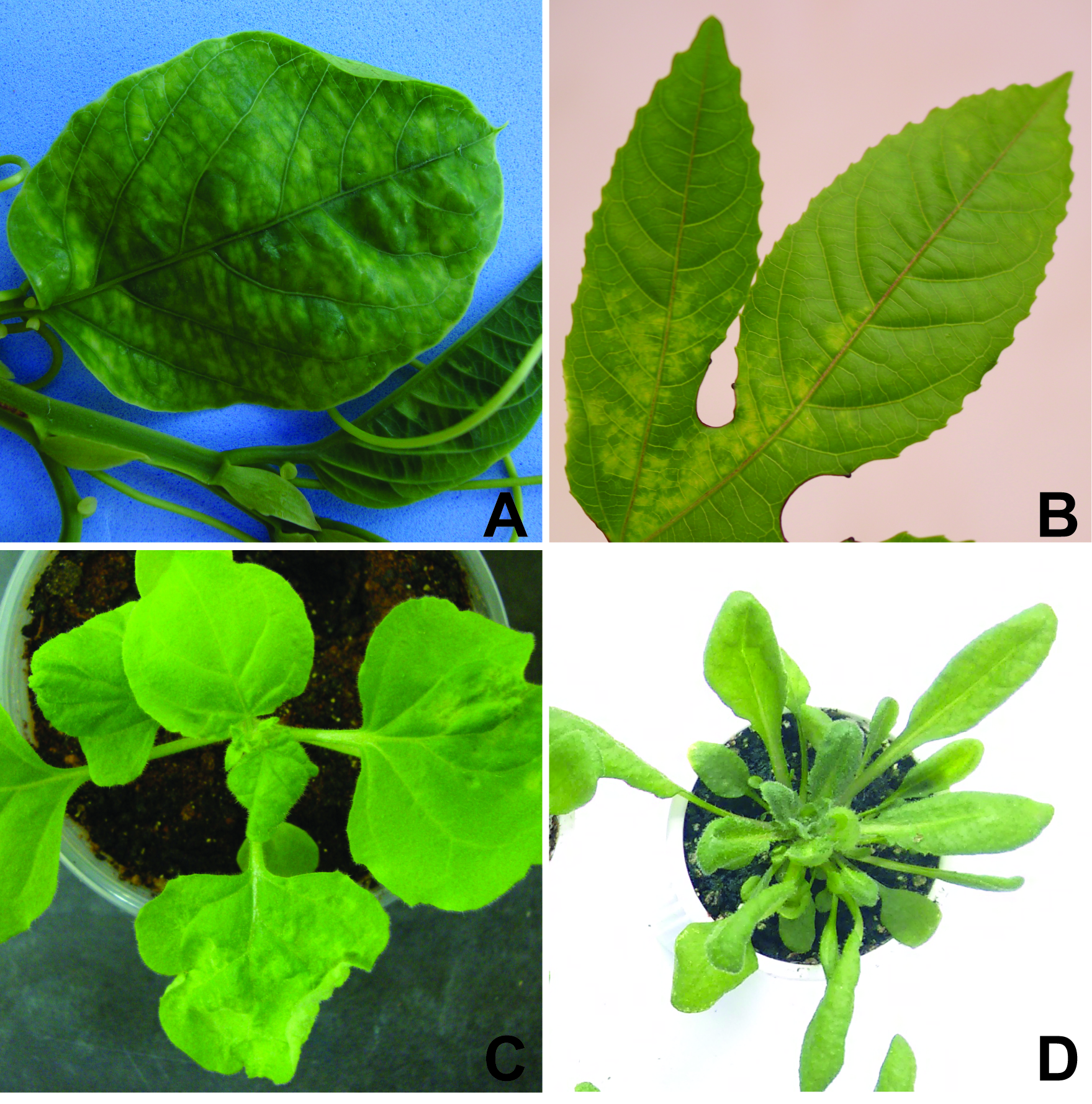

Supplement: Supplementary file 1 [file viruses-10-00169-s001.zip › Supplementary figure 1.tif]

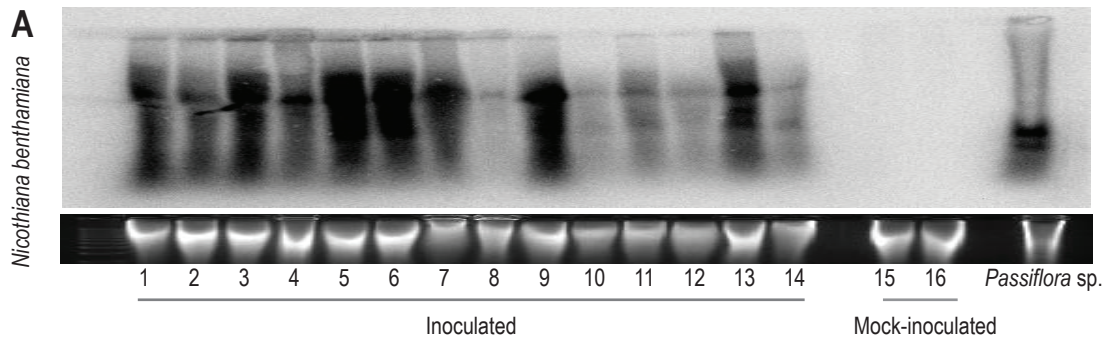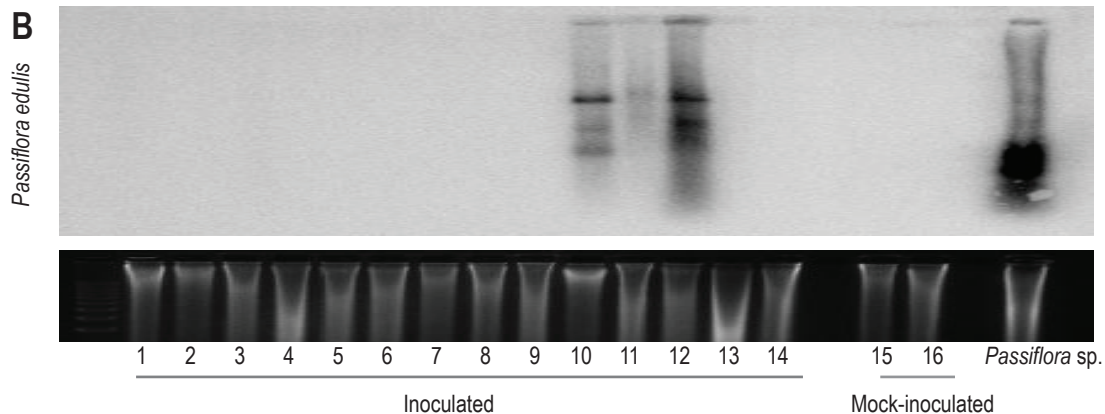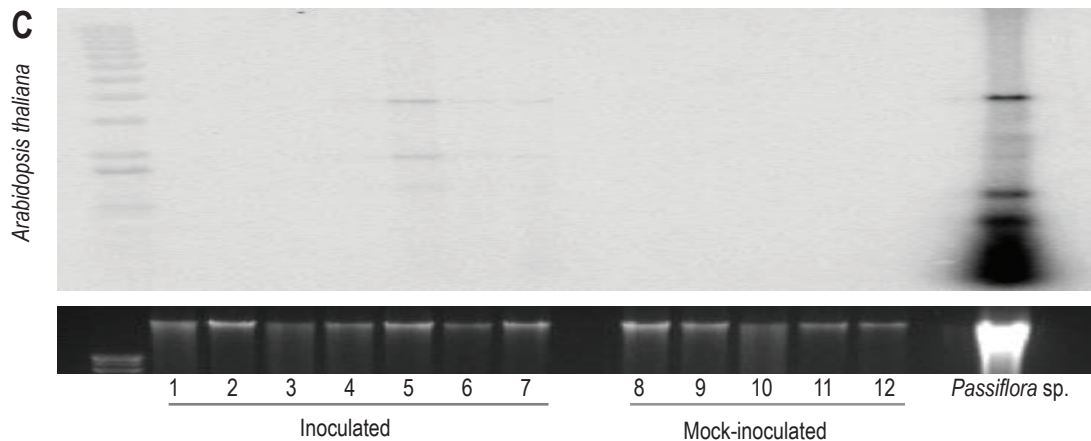

Supplement: Supplementary file 1 [file viruses-10-00169-s001.zip › Supplementary figure 2.pdf]
